# Clonazepam: An Old “New” Therapy for the Treatment of Phantom Limb Pain—A Brief Report of a Retrospective Study

**DOI:** 10.1155/2021/9966059

**Published:** 2021-09-27

**Authors:** Stefano Brunelli, Luca Pratesi, Marco Traballesi

**Affiliations:** Fondazione Santa Lucia, Scientific Institute for Research and Health Care, Rome, Italy

## Abstract

The purpose of this study is to describe the results of clonazepam use in the treatment of phantom limb pain (PLP). Although the efficacy of clonazepam on PLP has been reported in 1996, there are no subsequent known studies that confirmed this report. A consecutive sample of 32 patients who suffered from PLP after recent lower limb amputation was studied based on clinical charts. Wilcoxon's signed rank test was used to compare Numeric Rating Scale (NRS) values before and after the treatment with clonazepam. Twenty-three amputees were treated only with clonazepam, without adding other drugs or targeted rehabilitation treatments. The median NRS before the treatment with clonazepam was 7 (2), the median NRS after 31 ± 5 days of treatment was 3 (3.5) (*p* < 0.0001). The average dosage of clonazepam used was 1.5 ± 1 mg per day. The results suggest that clonazepam has to be considered as an alternative drug for PLP treatment.

## 1. Introduction

The phantom limb pain syndrome (PLP) is extremely common and affects up to 88% of people with limb loss [1] and about 14% of people with limb loss referred a PLP-related disability [2].

PLP is considered a chronic neuropathic pain syndrome, but its cause is still unknown [3]. Clinical findings suggest that both peripheral and central nervous system mechanisms can concur to the genesis of PLP [4].

Currently, there are no standard guidelines for the pharmacologic management of PLP. A Cochrane review of Alviar and coworkers reported not sufficient data to support any particular medication for established PLP [5]. The following review reported that no drug obtained a “level 1” of evidence for PLP relief while, limiting to reporting only to oral administration, “level 2” was reported for morphine, for an intermediate to long-term treatment effect, and for high dosage of gabapentin (2.400 mg per day) for a duration of 6 weeks [6].

Among the various pharmacologic agents that have been studied, a case report of 1996 described that clonazepam provided effective reduction in pain intensity in two amputees with PLP not responding to other drugs [7]. The authors stated that clonazepam has been omitted from journals and pain texts as a treatment option for PLP. And so, it has been until today!

In 25 years of clinical practice in rehabilitation and management of PLP in the Amputee Unit of our Institute for Research, Hospitalization and Health Care, the authors routinely use clonazepam oral administration. The aim of this study is to report a four-year retrospective analysis to describe the results of clonazepam use in the treatment of PLP.

## 2. Methods

This study analyses retrospectively the clinical charts of all patients consecutively admitted from the surgery unit after lower limb amputation for inpatient rehabilitation since 2015 to 2018. From the medical notes, the following epidemiologic characteristics and specific pathological data were recorded by manual collection: (1) age; (2) sex; (3) cause and (4) level of amputation; (5) days from amputation; and (6) days of recovery. Those people with limb loss already on drug treatment with clonazepam or with cognitive impairment or if their Numeric Rating Scale (NRS) data were missing were excluded from the study.

Analysis and discussion have been focused on these aspects: (1) how many patients suffered from PLP; (2) which drug was prescribed by surgery centers for the treatment of PLP; (3) how many patients we treated only with clonazepam and which dosage was used; (4) PLP evaluation before and after clonazepam treatment; (5) how many patients were not responders to clonazepam; and (6) side effects.

The PLP was measured using the 11-point interval scale NRS. As described in a previous work [8], patients were asked to rate their “average” pain felt in the last 7 days.

Wilcoxon's signed rank test was used to compare NRS values before and after the treatment with clonazepam. The alpha-level was set at 0.05 (two-tailed). Statisical analysis was performed with the use of R software, version 4.0.0.

Due to the negligible risk to patients, the local ethics committee stated that ethical approval was not required for this retrospective study. This study was performed in accordance with the Declaration of Helsinki.

### 2.1. Dosage Schedule of Clonazepam

According to our established PLP management routine, the treatment with clonazepam was started immediately at admission after the evaluation of pain. Simultaneously, a wash-out of the other drugs prescribed for PLP was started as stated by our common procedure.

Our progression pattern was to start with 5 drops (0.5 mg) once a day and then increasing to 3 drops each subsequent day (1 drop = 0.1 mg of active ingredient). The scheme provided increasing doses until the lowest effective dose. The maximum allowable dosage was 15 drops 3 times per day. If no pain reduction was obtained, we define these patients as “nonresponders” to clonazepam.

## 3. Results

In the reference years, 82 amputees were hospitalized. Of these, 39% (32 patients) suffered from PLP. At admission, 7 amputees were treated with gabapentin, 3 with NSAIDs, and one with pregabalin. After the wash-out, twenty-three amputees were treated with clonazepam as the only drug. Five amputees were treated with mirror therapy for research purposes and excluded from the analysis. Four patients were “not responders” to clonazepam and needed to combine other drugs. The demographic and clinical characteristics of the sample are summarized in Table 1.

The treatment period with clonazepam was 31 ± 5 days.  Table 2 summarizes NRS values at admission and at discharge for each patient. The improvement in NRS was statistically significant (*p* = 0.0001; *V* = 253).

The difference of the NRS means was greater than 1.0. This difference is considered as meaningful minimal clinically important difference [9].

As shown in Table 2, the highest dosage administered was 3.5 mg (10 drops in the morning + 10 at lunch time + 15 in the evening).

Side effects: twelve patients reported mild fatigue, drowsiness, or dizziness, but not so intense to reduce the dosage.

## 4. Discussion

This retrospective study has shown that the intensity of PLP could be reduced by clonazepam treatment. The mean NRS related to the PLP felt in last week significantly decreased from 7 to 3 in patients treated only with these drugs. Two patients reported the complete disappearance of the PLP. On the other hand, 4 amputees (14.8%) had no beneficial effects from clonazepam, even if, including NRS values of these patients in Wilcoxon's test analysis, the significance pre/postintervention is always <0.05 (not reported in the current brief report).

Clonazepam differs from other benzodiazepines because it binds more to central than to peripheral benzodiazepine receptor sites and, possibly for this reason, has shown to be effective in the treatment of a chronic pain syndrome as the “burning mouth syndrome” [10].

The exact mechanism by which clonazepam exerts its effects is unknown. An emerging hypothesis suggests that PLP may depend by structural changes in the corpus callosum of people with limb loss that determines a decreased cortical inhibition mediated through excitatory callosal neurons, which act on local GABAergic neurons [11]. The effect of clonazepam may be explained through its agonistic action at the inhibitory GABA-A receptor. Another hypothesis is clonazepam effects on the suppression of the spontaneous central neuronal hyperactivity that occurs after deafferentation [12].

The effect of clonazepam was studied only in amputees with a mean interval from surgery of about one month. A physiological decrease of PLP intensity cannot be excluded. The type of this study (retrospective case series) did not allow a comparison with a control group. Another limitation is the lack of control for confounding variables such as prosthesis use, demographic factors, or comorbidities.

Clonazepam produces positive effects reducing PLP intensity and could be used as an alternative treatment. It is apparently a safe treatment with low adverse effects. Further studies are necessary to confirm the efficacy of clonazepam on PLP reduction.

## Figures and Tables

**Table 1:** Demographic and clinical characteristics of the sample.

Medical notes	Patient data
*N* (male/female)	16/7 (m 69.5%)
Age (mean ± SD)	61.2 ± 14.5
Level (TT/TF)	9/14 (TF 68.9%)
Cause of amputation (Tr/Dy)	7/16 (dysvascular 69.5%)
Time interval from amputation	31.4 ± 11.3 days

TT: transtibial; TF: transfemoral; Tr: trauma; Dy: dysvascular.

**Table 2:** NRS admission: pain score before the treatment with clonazepam. NRS discharge: pain score after the treatment with clonazepam. ^∗^*p* < 0.005. The reported averages are median (interquartile) for NRS values and mean (SD) for clonazepam dosages.

Patients	NRS admission	NRS discharge	Clonazepam mg
1	9	3	2.5
2	6	2	0.5
3	8	3	1.3
4	4	0	0.3
5	5	1	0.5
6	6	3	0.3
7	8	2	0.6
8	6	3	0.8
9	9	5	3.5
10	6	0	0.6
11	8	3	1.5
12	7	7	2.5
13	10	6	3.5
14	8	6	1.8
15	7	6	1.5
16	8	7	2.5
17	6	4	2
18	9	3	2
19	8	5	1.5
20	5	3	0.5
21	7	1	1
22	10	7	2.5
23	5	0	1
*Averages*	7 (2)	3 (3.5)^∗^	1.5 ± 1

## Data Availability

The data supporting the results of this study can be requested to the corresponding authors.
